# β-micrustoxin (Mlx-9), a PLA_2_ from *Micrurus lemniscatus* snake venom: biochemical characterization and anti-proliferative effect mediated by p53

**DOI:** 10.1590/1678-9199-JVATITD-2021-0094

**Published:** 2022-04-11

**Authors:** Natália Fernanda Teixeira dos Santos, Andréia de Souza Imberg, Douglas Oscar Ceolin Mariano, Angelina Cirelli de Moraes, Jessica Andrade-Silva, Cristina Maria Fernandes, Ana Cláudia Sobral, Karina Cristina Giannotti, Wilson M. Tatagiba Kuwabara, Daniel Carvalho Pimenta, Durvanei Augusto Maria, Maria Regina Lopes Sandoval, Solange Castro Afeche

**Affiliations:** 1 Laboratory of Pharmacology, Butantan Institute, São Paulo, SP, Brazil.; 2 Laboratory of Biochemistry and Biophysics, Butantan Institute, São Paulo, SP, Brazil.; 3 Laboratory of Molecular Biology, Butantan Institute, São Paulo, SP, Brazil.; 4 Department of Physiology and Biophysics, Institute of Biomedical Sciences, University of São Paulo (USP), São Paulo, SP, Brazil.; 5 Contaminants Center, Water and Packaging Core, Adolfo Lutz Institute, São Paulo, SP, Brazil.; 6 Special Pain and Signaling Laboratory, Butantan Institute, São Paulo, SP, Brazil.

**Keywords:** Astrocytes, Glioblastoma, β-micrustoxin, Mlx-9, PLA_2_, *Micrurus lemniscatus* venom, p53 protein

## Abstract

**Background:**

Endogenous phospholipases A_2_ (PLA_2_) play a fundamental role in inflammation, neurodegenerative diseases, apoptosis and cellular senescence. Neurotoxins with PLA_2_ activity are found in snake venoms from the Elapidae and Viperidae families. The mechanism of action of these neurotoxins have been studied using hippocampal and cerebellar neuronal cultures showing [Ca^2+^]i increase, mitochondrial depolarization and cell death. Astrocytes are rarely used as a model, despite being modulators at the synapses and responsible for homeostasis and defense in the central nervous system. Preserving the cell division ability, they can be utilized to study the cell proliferation process. In the present work cultured astrocytes and glioblastoma cells were employed to characterize the action of β-micrustoxin (previously named Mlx-9), a PLA_2_ isolated from *Micrurus lemniscatus* snake venom. The β-micrustoxin structure was determined and the cell proliferation, cell cycle phases and the regulatory proteins p53, p21 and p27 were investigated.

**Methods:**

β-micrustoxin was characterized biochemically by a proteomic approach. Astrocytes were obtained by dissociation of pineal glands from Wistar rats; glioblastoma tumor cells were purchased from ATCC and Sigma and cultured in DMEM medium. Cell viability was evaluated by MTT assay; cell proliferation and cell cycle phases were analyzed by flow cytometry; p53, p21 and p27 proteins were studied by western blotting and immunocytochemistry.

**Results:**

Proteomic analysis revealed fragments on β-micrustoxin that aligned with a PLA_2_ from *Micrurus lemniscatus lemniscatus* previously identified as transcript ID DN112835_C3_g9_i1/m.9019*.* β-micrustoxin impaired the viability of astrocytes and glioblastoma tumor cells. There was a reduction in cell proliferation, an increase in G2/M phase and activation of p53, p21 and p27 proteins in astrocytes.

**Conclusion:**

These findings indicate that β-micrustoxin from *Micrurus lemniscatus* venom could inhibit cell proliferation through p53, p21 and p27 activation thus imposing cell cycle arrest at the checkpoint G2/M.

## Background

Endogenous phospholipases A2 (PLA_2_s) play a fundamental role in inflammation, neurodegenerative disorders, apoptosis, cancer and cellular senescence. PLA_2_s are classified according to their functional and structural features while the most important ones are part of the secretory (sPLA_2_), cytosolic and Ca^2+^-independent families [1, 2]. 

The sPLA_2_s are found in animal venoms, particularly in the snake venoms from the Elapidae and Viperidae families [3]. These snake venoms sPLA_2_s have a fundamental role in the symptoms observed after snake bites and are named β-neurotoxins because they exhibit presynaptic activity at motor neurons causing the massive release of acetylcholine. The β-neurotoxins together with α-neurotoxins, a three-finger class of toxins that antagonize muscle cholinergic receptors [4-6], act in synergy at the neuromuscular junction inducing flaccid paralysis in humans and snakes’ preys, thus being important determinants of death by respiratory paralysis [7-12]. 

PLA_2_s (EC 3.1.1.4) are enzymes that catalyze the hydrolysis of the *sn*-2 ester bond of glycerophospholipids producing lysophospholipids and free fatty acids. The sPLA_2_s are proteins of low molecular mass, between 13-19 kDa, with five to eight disulfide bridges that require calcium for the catalytic activity [13-16]. These sPLA_2_s are grouped into 15 classes among which those from Elapidae and Viperidae snake venoms belong, respectively, to the classes IA and IIA [3, 17]. 

Enzymatic activity of β-neurotoxins is responsible for important pathophysiological changes in the plasma membrane that lead to massive release of neurotransmitters and impaired recycling of synaptic vesicles [14, 15]. Although this hydrolytic activity has an important role in the toxin’s effects, it does not explain all the specific outcomes found in different cell types. In view of this fact, it was proposed that PLA_2_ toxins could bind to different membrane receptors thus allowing the entry of the toxin into cells. Supporting this proposition is the fact that PLA_2_ toxins are found inside neurons and even co-localized with the nucleus or mitochondria [18-20]. Moreover, the binding of sPLA_2_ to the PLA_2_ receptor (PLA_2_R) triggers intracellular signaling cascades, especially those involved with apoptosis, proliferation and senescence [21]. Thus, the entire complexity of the mechanisms of action of these sPLA_2_ toxins is not completely clear. 

It was reported that sPLA_2_ could induce cell proliferation in different cell types such as fibroblasts, lymphoblastoid cells and astrocytoma [22-24]. Also, the occurrence of senescence involving sPLA_2_, the PLA_2_R and the mediation of p53 protein has been demonstrated [25-28]. Another important finding related to sPLA_2_ is its antitumor activity, and toxins that exhibited anticancer effects were isolated from the snake venoms of the genus *Bothrops*, *Crotalus* and *Naja* [29-33]. These discoveries turn these enzymes into potential targets of anticancer therapeutic agents. VRCTC-310-Onco, an antitumor pharmaceutical product composed of crotoxin and cardiotoxin, sPLA_2_s from *Crotalus durissus terrificus* and *Naja naja atra* respectively, interferes with the epidermal growth factor signaling [29].

Cultured neurons from the central nervous system, such as hippocampal and cerebellar granule neurons, have been successfully used as a model to characterize the mechanisms of action of β-neurotoxins. It was reported that β-neurotoxins - including β-bungarotoxin, textilotoxin, taipoxin, notexin, Mlx-8 and Mlx-9 (respectively from the venoms of *Bungarus multicinctus, Pseudonaja textilis*, *Oxyuranus scutellatus, Notechis scutatus* and *Micrurus lemniscatus*) - increase intracellular Ca^2+^ ([Ca2+]i), depolarize mitochondrial membrane, generate reactive oxygen species and induce cell death in cultured neurons [34-38]. On the other hand, glial cells, despite playing important roles in the central and peripheral nervous system, are poorly used [18, 35]. Astrocytes are the predominant glial cells composing the tripartite synapse, acting as modulators by releasing gliotransmitters such as TNF-α, D-serine and ATP, and participating in homeostasis, neurotransmitters recapture, extracellular K^+^ ion equilibrium and neuroprotection [39-44]. In the present work, we used astrocytes to analyze a cell proliferation process.

From the venom of the South American coral snake *Micrurus lemniscatus*, four toxins with PLA_2_ activity were previously isolated [45]. *In vivo* and *in vitro* studies on two of these sPLA_2_ toxins, Mlx-8 and Mlx-9, demonstrated that both induced electroencephalographic and behavioral alterations after *in vivo* intrahippocampal administration, and apoptosis and necrosis in cultured hippocampal neurons [34, 45]. In the present study, Mlx-9, now termed β-micrustoxin, was proteomically characterized and its actions on viability and proliferation process were analyzed in astrocytes and in U138 and U251 glioblastoma tumor cells that originate from astrocytes. The previously given name Mlx-9 comes from the fact that it was eluted at peak 9 of the chromatogram of *Micrurus lemniscatus* venom [34, 45]. The new name β-micrustoxin is due to its action on the neuronal terminal and the provenance from *Micrurus lemniscatus* venom. A possible interference with the distribution of cell cycle phases, p53, p21 and p27 regulatory proteins, as well as TNF-α release was investigated in cultured astrocytes. As cultured astrocytes are proliferating cells, they constitute a good model for studies of the cell proliferation process and of cell cycle regulators and disruptors from animal venoms.

## Methods

### Animals

Male Wistar rats (200-250g) were kept under a 12h:12h light-dark cycle, in a temperature-controlled room (21 ± 2°C) with water and food *ad libitum*. This research was conducted in accordance with the Ethical Principles in Animal Research, adopted by the Brazilian College of Animal Experimentation, and was approved by the Ethics Committee for Animal Research of Butantan Institute (Protocol number 1223/14). 

### Venom

*Micrurus lemniscatus* crude venom was kindly provided by the Laboratory of Venomous Animals, Federal University of Bahia, Brazil. It was lyophilized and stored at -20°C until use. 

### RP-HPLC purification of β-micrustoxin

β-micrustoxin was isolated by reversed-phase high performance liquid chromatography as described elsewhere [45]. Briefly, *M. lemniscatus* venom (10 mg) was diluted in 5 mL of Milli-Q water. After filtration using a 0.45 µm filter (Millipore), 400 µL samples (800 µg) were applied to a C8 reversed-phase column (Shim-Pack; 4.6 mm x 250 mm, 5 µm particle) coupled to a HP 1100 series HPLC system. The elution was carried out under a flow rate of 1 mL/min and monitored at 214 nm. A linear gradient of trifluoroacetic acid (TFA) (solvent A) (0.1% TFA in water) and acetonitrile (solvent B) (90% acetonitrile + 10% solvent A), from 10% to 35% of solvent B in 50 min, was utilized to elute proteins. Peaks were manually collected according to the optical density. Peak 9 corresponded to β-micrustoxin [45]. β-micrustoxin concentrations used in the present work were chosen based on Carvalho *et al*. [34]. 

### Biochemical characterization of β-micrustoxin

Mass spectrometry analysis

β-micrustoxin aliquots were dried (± 5 µg for each sample) and submitted to in-solution digestion according to Beraldo-Neto *et al.* [46]. Each sample was resuspended in 8 M urea (in 100 mM Tris-HCL, pH 8.5) and Tris (2-carboxyethyl) phosphine hydrochloride (TCEP) (dissolved in water, 20 mM final concentration) and the mixtures were kept for 1 h at room temperature. Subsequently, iodoacetamide (IAA) (dissolved in water, 10 mM final concentration) was added and the mixture was kept for 1 h at room temperature and protected from light. Next, 100 mM Tris-HCl (pH 8.5) was added to dilute urea concentration to ≤ 2 M, and furthermore 10 µL chymotrypsin (40 ng.µL^-1^ in 100 mM Tris-HCl, pH 8.5) or 10 µL trypsin (10 ng.µL^-1^ in 100 mM Tris-HCl, pH 8.5) was added. Digestion was carried out for 18 h, at 37°C. Finally, 50% acetonitrile/5% TFA was added to stop the enzymatic reaction. All samples were dried. Prior to mass spectrometer analysis, each β-micrustoxin sample was desalted and the peptides were concentrated by ZipTip® C-18 pipette tips (Millipore). The ZipTip® C-18 step was repeated twice; the concentrated material of each sample was pooled and dried.

Each dried β-micrustoxin sample was resuspended in 0.1% acetic acid and analyzed by liquid chromatography-mass spectrometry (LC-MS) using an electrospray-ion-trap time-of-flight (ESI-IT-TOF) system coupled to a binary ultra-fast liquid chromatography system (UFLC) (20A Prominence, Shimadzu, Kyoto, Japan). All samples were loaded into a C18 column (Discovery C18, 5 μm, 50 × 2.1 mm) operating with a binary solvent system: (A) water: acetic acid (999:1, v/v) and (B) acetonitrile: water: acetic acid (900:99:1, v/v/v). The column was eluted at a constant flow rate of 0.2 mL/min with a 0 to 40% linear gradient of solvent B for 35 min. The eluates were monitored by a Shimadzu SPD-M20A PDA detector before being introduced into the mass spectrometer. The interface voltage was set to 4.5 kV; the capillary voltage was 1.8 kV at 200°C, and the fragmentation was induced by argon collision at 50% energy. The MS spectra were acquired under the positive mode and collected in the range of 390 to 1400 m/z. The MS/MS spectra collected ranged from 50 to 1950 m/z. 

Data processing and data analysis

LCD Shimadzu raw data were converted to mascot generic format (MGF) files using the software LCMS Protein Postrun (Shimadzu, Kyoto, Japan) and loaded in the software Peaks Studio V7.0 (Bioinformatics Solutions Inc, BSI, Waterloo-ON, Canada) [47]. Proteomic identification was performed according to the following parameters: error mass (MS and MS/MS) was set to 0.2 Da; methionine oxidation and carbamidomethylation were set as variable and fixed modifications, respectively; chymotrypsin or trypsin was used as proteolytic enzyme for cleavage; maximum missed cleavages (3), maximum variable PTMs per peptide (3), and non-specific cleavage (one) were determined. Data were analyzed against a database composed of the venom gland proteome (68164 entries) obtained from 6 *Micrurus* taxa (National Center for Biotechnological Information - NCBI - BioProject: PRJDB5628; ID: 383534) and some new phospholipase A2 sequences (6 entries added manually in the database) identified by Aird *et al.* [7] (Figure 12A of the respective paper). All identified sequences were aligned using a basic local alignment search for proteins (BLASTp), limiting the search to the order Squamata (taxid: 8509) [48]. Thus, an alignment with a higher score was achieved in this study.

### Astrocyte culture

Astrocytes were obtained from pineal glands by papain digestion (Papain Dissociation System, Worthington Biochemical Corporation, Freehold, NJ, USA), according to the method described by Villela *et al*. [49]. For each procedure eight glands were isolated and immediately placed in ice-cold Dulbecco’s Modified Eagle’s Medium (DMEM) (glucose: 1000 mg/L, HEPES: 5.9 g, sodium bicarbonate: 3.7 g) (Sigma, St. Louis, MO, USA). Then, the tissue was incubated at 37 °C for 45 minutes in papain (0.01%) and DNase (0.01%) solution. After papain removal and blockade with ovomucoid (2 mg/mL), the cells were mechanically dispersed and resuspended in DMEM supplemented with 10% fetal calf serum (FCS) and 1% penicillin-streptomycin. Cells were cultivated in 75 cm^2^ culture flasks, at 37°C, in 5% CO_2_, for 16 h. Culture medium with free-floating pinealocytes was removed; the astrocytes remained attached to the culture flasks and were kept in culture for one week. After this period, two cell passages were performed as follows: cells were washed twice with Hanks’s solution and then 6 mL of 0.25% trypsin was added for 5 min. Trypsin action was blocked with DMEM supplemented with 10% FCS. Cells were then centrifuged at 300g for 5 min at 20(C. The pellet was suspended at a concentration suitable for the required assay.

### U138 and U251 glioblastomas cell culture

U138 (ATCC® HTB-16) and U251 (Sigma - 09063001) human glioblastoma tumor cells were acquired, respectively, from American Type Culture Collection (ATCC, Manassas, VA, USA) and Sigma (Darmstadt, Germany) and stored in liquid nitrogen. These cells were cultivated in DMEM medium supplemented with 10% FCS and 1% penicillin-streptomycin. Cell passages were obtained as described for astrocytes (see “Astrocyte culture” section).

### Cell viability assay

Astrocytes or glioblastoma tumor cells (2 x 10^4^ cell/well) were cultivated in DMEM medium with 10% FCS in 96-well plates at 37°C, in 5% CO_2_ for 24 h before the treatments. Cells were incubated in the presence or absence (control) of β-micrustoxin (0.2, 2, 20, 200 nM) for 3, 12 or 24 h (only 24 h for glioblastomas) and 250 mM KCl was used as a positive control. Next, the medium was altered by DMEM without FCS (90 µL) and 10 µL MTT [3-(4,5-dimethylthiazol-2-yl)-2,5-diphenyltetrazolium bromide] solution (5 mg/mL in PBS), and the cells remained for 3 h at 37°C, in 5% CO_2_. Subsequently, 100 µL DMSO was added to each well to solubilize the formed crystals and the plate was agitated for 30 min. Absorbance was evaluated at 570 nm. The results were expressed as a percentage in relation to the control group.

### Cell proliferation assay

Cell proliferation was evaluated by labeling the cells with CFSE fluorophore (Carboxyfluorescein diacetate succinimidyl ester - Molecular Probes, ThermoFisher Scientific, MA, USA). CFSE labels both resting and proliferating cells and equally spreads to daughter cells upon cytokinesis both *in vitro* and *in vivo*. The CSFE method allows for direct detection of single proliferating cells and facilitates quantification of cell division by flow cytometry [50, 51]. 

Astrocytes (6 x 10^5^ cells/well) were cultivated in 6-well plates in DMEM medium with 10% FCS at 37°C, in 5% CO_2_ and treated for 24 h with 5 µM CSFE diluted in PBS and 0,1% human albumin. Cells were incubated in the presence or absence (control) of β-micrustoxin (0.2, 2, 20, 200 nM) for 24 h. After treatment, the cells were collected by trypsinization, washed 3x with 20 mL DMEM and 10% FCS, centrifuged at 300g and finally suspended in 1 mL DMEM. The results were obtained by flow cytometry (Facscalibur- Becton Dickson) and analyzed by the program FlowJo using the index of cellular division. This index represents the mean number of cell divisions not considering the cells that remained in the zero generation. 

### Evaluation of cell cycle phases

Astrocytes were cultivated (6 x 10^5^ cells/well) in 6-well plates in DMEM medium with 10% FCS at 37°C, in 5% CO_2_ for 24 h. Next, they were incubated in the presence or absence (control) of β-micrustoxin (0.2, 2, 20, 200 nM) for 24 h. Cells were collected by trypsinization and suspended in PBS, centrifuged 2x at 300g and suspended in 200 µL propidium iodide (Sigma-Aldrich, St. Louis, MO, USA) (18 μg) solution with 20 uL Triton X-100 and 4 mg RNAse A for 30 min at room temperature in the dark. The images were collected using flow cytometry (Facscalibur- Becton Dickson). Cell cycle phases (G0/G1, S and G2/M) and sub-G1 population were expressed as a percentage.

### Analysis of p53, p21 and p27 regulatory proteins

Analysis of p53, p21 and p27 by flow cytometry

Astrocytes were cultivated and treated in the same conditions described in “Evaluation of cell cycle phases” section, except that the treatment duration was 2 h or 6 h. Then cells were collected by trypsinization and fixed with 4% paraformaldehyde for 1 h, at 4°C, with 1 µg of specific antibodies: p53 (mouse - ab26), p21 (rabbit - ab7960) and p27 (rabbit - ab32034). Next, cells were centrifuged at 300g and washed with cold PBS. The supernatant was discarded, and the cells were suspended in Facs buffer (Beckman-Coulter). Proteins expressions were evaluated using flow cytometry (FACScalibur, Becton Dickson) in FL-1 fluorescence intensity and analyzed by the program Cell-Quest (BD).

Analysis of p53, p21 and p27 by western blotting

Astrocytes (2.5 x 10^5^ cells/well) were seeded in 24-well plates in DMEM + 10% FCS medium, at 37ºC in 5% CO_2_ for 24 h. Subsequently, they were incubated in culture medium only (control) or in culture medium with β-micrustoxin (2 or 20 nM) for 2 h. Cells were lysed by the addition of RIPA buffer with a protease inhibitor cocktail (Roche Diagnostsics) and 10% 0.1 M phenylmethylsulfonyl fluoride (PMSF). Protein content was determined in the supernatant of tissue extracts using a BCA kit (Thermo Scientific, Rockford, IL, USA), and Laemmli Sample Buffer was added to the samples. Aliquots of these samples (20 μg/well) were subjected to electrophoresis for separation by SDS-PAGE in 4-20% Criterion^TM^ TGX^TM^ precast gels (Bio-Rad, CA, USA) (120 V, 1 h) and electrophoretically transferred to nitrocellulose membranes using the transfer kit and FASTBLOT equipment (BioRad), according to the manufacturer's instructions. Membranes were blocked for 1 h at room temperature with 5% BSA in TBST [20 mM Tris - HC1 (pH 7.6) containing 150 mM NaCl and 0.1% (v/v) Tween 20]. Next, membranes were incubated overnight, at 4°C, with the following primary antibodies: p21 (Abcam; ab109199), p27 (Abcam; ab32034) or p53 (Abcam; ab26). Membranes were then incubated with the secondary antibodies IRDye 800CW or IRDye 680CW (1: 10,000, Li-COR) in TBST for 45 min at room temperature. Fluorescence of the bands was detected using ODYSSEY equipment (LI-COR Biosciences), blots were normalized by Ponceau analysis, and the optical density of the bands was determined using the software Image J (NIH).

Immunocytochemistry for p53

Astrocytes (2x10^4^ cells/well), adhering to 13 mm glass coverslips, were cultured in DMEM + 10% FCS, at 37ºC, 5% CO_2_. Cells were treated with either 2 nM β-micrustoxin, or 1 mM cyclophosphamide, or 1 µM doxorubicin or 1 µM gemcitabine (the latter three are antitumor drugs that were used as positive controls) for 24 h. After this period, the coverslips were washed twice with Hank's balanced solution (HBSS), without calcium, magnesium and phenol red (Gibco). Next, cells were fixed with 2% paraformaldehyde for 30 min, at room temperature. The coverslips were washed 3 times with HBSS. After permeabilization and blocking of nonspecific sites with 0.1 M buffer solution + 0.5% albumin + 0.2% triton, for 30 min, at room temperature, the coverslips were washed again. Subsequently, cells were incubated with a primary anti-p53 antibody solution (ab26, Abcam) at a 1: 150 dilution overnight at 4°C. The coverslips were washed with HBSS and incubated with a secondary Alexa 488 anti-mouse antibody solution (1: 300) (A-11017, Invitrogen) containing Hoechst (2: 5000) (33342, Thermofisher) for 1 h, at room temperature. Cells were washed with HBSS and the coverslips were mounted on microscope slides with 10 µL of the anti-fading Fluoromount-G (00-4958-02, Invitrogen). Images were obtained by a Leica DMi8 confocal microscope (TCS SP8, Leica), by the software LAS X at 63x magnification.

### TNF-α evaluation

To assess the involvement of TNF-α in the toxin’s effects on cell proliferation, the same procedure described in “Cell proliferation assay” section was followed. Cells were incubated in culture medium (at the same volume as the experimental groups) in the absence (control) or presence of 9 ng/mL recombinant TNF-α, or 2 nM β-micrustoxin, or 2 nM β-micrustoxin + 2 µg/mL anti-TNF-α for 24 h and then washed 3 times with DMEM medium and 10% FCS, centrifuged and suspended in 1 mL of the same medium. Data analysis was performed as described in “Cell proliferation assay”.

To quantify the TNF-α released, astrocytes were seeded in 6-well plates (6x10^5^ cells/well) and incubated with culture medium (same volume as the experimental groups) in the absence (control) or presence of β-micrustoxin (2 or 20 nM), or β-micrustoxin (2 or 20 nM) + 2 µg/mL anti-TNF-α, or 9 ng/mL recombinant TNF-α or 9 ng/mL recombinant TNF-α + 2 µg/mL anti-TNF-α for 5 h or 24 h, at 37ºC, 5% CO_2_. Subsequently, the medium was collected, centrifuged for 5 min at 16,000g (Eppendorf 5415C, Brinkman Instruments Inc., Westburg, N.Y., USA) and stored in a freezer at -80°C. These supernatants were tested for the presence of TNF-α using the cytotoxicity assay on fibroblast continuous cell line L929 [52-54]. Briefly, these cells were cultured in microtiter plates (3.5 x 10^4^ cells/well), in RPMI medium, with 40 µg/mL gentamicin, 2 mM L-glutamine and 10% FCS, and incubated for 24 h at 37°C in 5% CO_2_. The astrocyte supernatants were serially diluted in RPMI-1640 containing 1.0 mg/mL of actinomycin D. Two hundred µL of each dilution was added to the cells and assayed in triplicate experiments. After incubation for 20 h at 37ºC, supernatants were removed and viable cells were assessed after staining with crystal violet (0.5% in 30% acetic acid). Absorbance was determined at 620 nm. The cytotoxicity percentage was calculated as follows: (Abs control - Abs sample/Abs control) x 100. TNF-α levels were then expressed as U/mL using a standard curve prepared with recombinant TNF-α.

### Statistical analysis

Data were expressed as mean ± SEM. The analysis was accomplished by one-way or two-way ANOVA followed by Bonferroni *post-hoc* test using Prism 7.0. Significance was defined as *p* < 0.05.

## Results

### Biochemical characterization of β-micrustoxin by proteomic analysis

**Figure 1.**
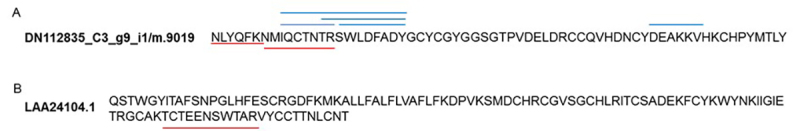
Protein sequences identified in β-micrustoxin. The β-micrustoxin was submitted to an in-solution digestion by chymotrypsin or trypsin enzyme and the proteomic analysis was performed against a proteome database obtained from the venom gland of six *Micrurus* (coral snakes) taxa. The chymotryptic (blue; upper-line) or tryptic (red; underline) peptides were highlighted. Two protein sequences were identified: **(A)** the transcript ID DN112835_C3_g9_i1/m.9019 and the sequence **(B)** LAA24104.

Using the strategy of digesting the β-micrustoxin sample with two different proteolytic enzymes, respectively chymotrypsin and trypsin, the present proteomic study identified the presence of two proteins in this fraction: the PLA_2_ transcript ID DN112835_C3_g9_i1/m.9019 from *Micrurus lemniscatus lemniscatus* (Figure 1A), described in the work of Aird *et al.* [7] and the hypothetical protein LAA24104 from *Micrurus lemniscatus carvalhoi* [7] (Figure 1B). Seven peptides identified in β-micrustoxin aligned with the first sequence (Table 1; Additional files 1 and 2), while only one peptide aligned with the second one (LAA24104) (Table 1; Additional file 3). Previously, a single protein with PLA_2_ activity was identified in the fraction of β-micrustoxin by mass spectrometry showing the predominance of this protein in the fraction under study [45].

**Figure 2.**
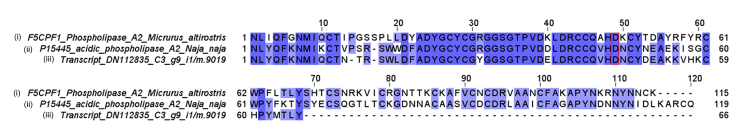
The sequence of two known snake venom phospholipases, (i) phospholipase A2 (*M. altirostris* - UniProtKB F5CPF1) and (ii) acidic phospholipase A2 (*N. naja* - UniProtKB P15445), were aligned against the identified protein sequence (iii) transcript ID DN112835_C3_g9_i1/m.9019 using Clustal O (1.2.4) multiple sequence alignment. The blue scale color showed the similarity between each sequence. Dark blue: all sequences have the same amino acid in this position; light blue: two sequences have the same amino acid in this position. It is possible to observe the similarity between the sequence DN112835_C3_g9_i1/m.9019 and the other two phospholipase sequences. Aspartate at position 49, which is characteristic of a PLA_2_, is highlighted.

After the proteins identification, a BLASTp analysis was performed against the Squamata database. The PLA_2_ transcript ID DN112835_C3_g9_i1/m.9019 has a higher similarity to several Elapidae venom phospholipases, such as the PLA_2_ from *M. altirostris* (UniProtKB F5CPF1) and from *N. naja* (UniProtKB: P15445) (Figure 2). Furthermore, it is worth pointing out the presence of the aspartate at position 49, which is characteristic of a PLA_2_. Unfortunately, the identified PLA_2_ protein (transcript ID DN112835_C3_g9_i1/m.9019) appears to be incomplete. 

After further BLASTp analysis, the second identified protein (denominated hypothetical protein LAA24104) aligned with a putative three-finger toxin present in *M. altirostris* venom (UniProtKB: AED89561) (data not shown). 

**Table 1.** List of proteins identified in β-micrustoxin by proteomic analysis against *Micrurus* protein database.

Enzyme	Transcript ID	Peptides	Organism	Mass (Da)	Length	Mass/charge (m/z)	z
Chymotrypsin	DN112835_g9_i1/m9019	M.IQC(+57.02)*TNTRSWLDFADY M.IQC(+57.02)*TNTR R.SWLDFADY N.TRSWLDFADY Y.DEAKKVH	*M. lemniscatus lemniscatus*	1,888.8414	15	945.4262	2
				891.4233	7	446.7125	2
				1,015.4287	8	1.016.4211	1
				1,272.5775	10	637.3131	2
				825.4344	7	826.4419	1
Trypsin	DN112835_g9_i1/m9019	NLYQFKK.NMIQCTNTR	*M. lemniscatus lemniscatus*	811.4228	6	812.4644	1
	LAA24104.1	K.TCTEENSWTAR		1,136.5067	9	569.2442	2
			*M. lemniscatus carvalhoi*	1.353,56	11	678	2

### Effects of β-micrustoxin on cell viability of astrocytes and glioblastoma cells

**Figure 3.**
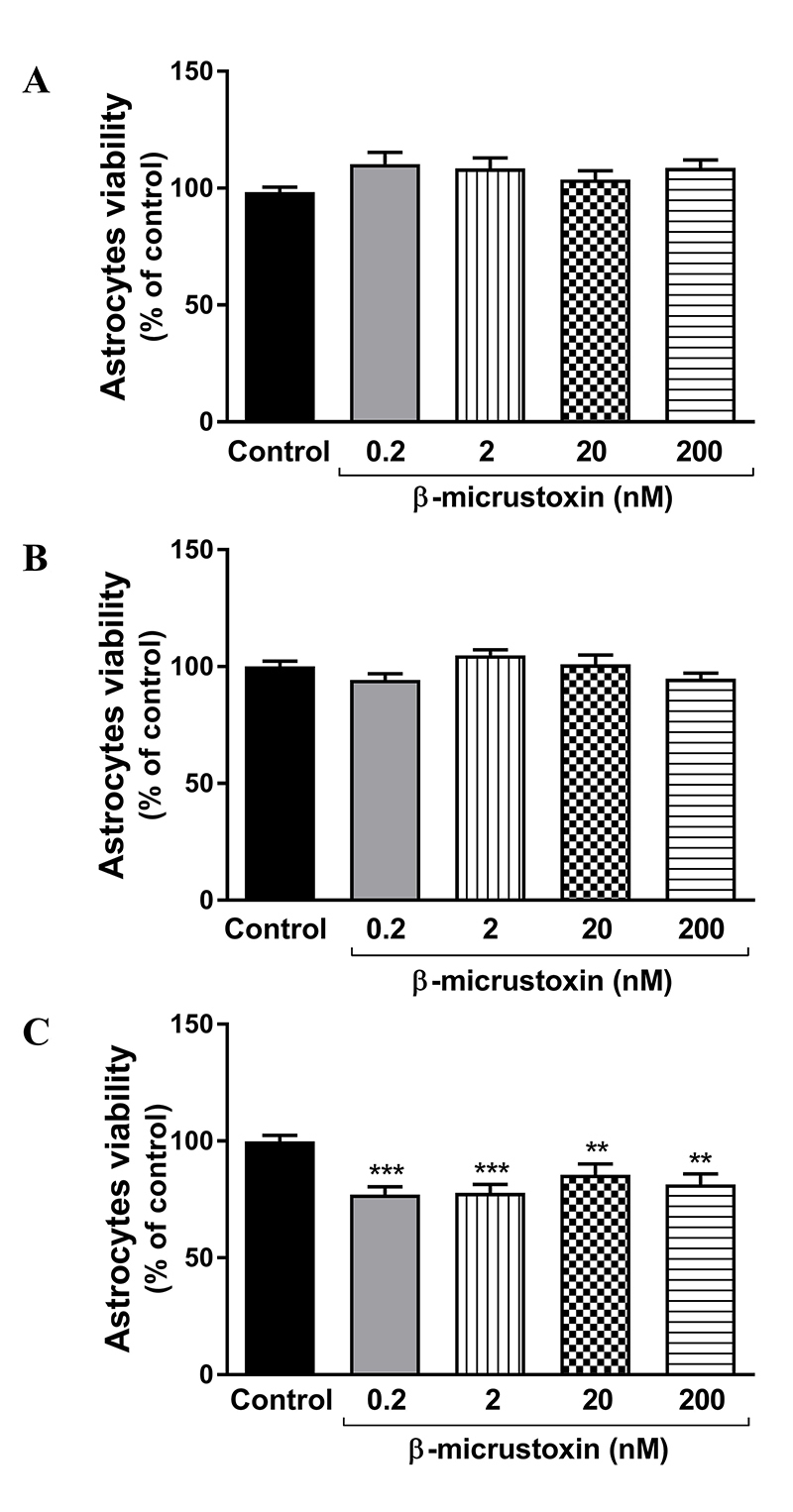
Viability of cultured astrocytes incubated in the absence (control) or presence of β-micrustoxin (0.2, 2, 20, 200 nM) for **(A)** 3 h, **(B)** 12 h, **(C)** 24 h. N = 20/group. One-way ANOVA followed by Bonferroni *post-hoc* test: **p < 0.01; ***p < 0.001 vs. control, three full experimental blocks.

β-micrustoxin reduced cell viability of cultured astrocytes after 24 h of incubation for all the concentrations used (control = 100 ± 2.59%; 0.2 nM toxin = 77.1 ± 3.35%; 2 nM toxin = 77.87 ± 3.53%; 20 nM toxin = 85.54 ± 4.67%; 200 nM toxin = 81.48 ± 4.51%). The same effect was not observed after 3 h or 12 h of toxin incubation (Figure 3).

**Figure 4.**
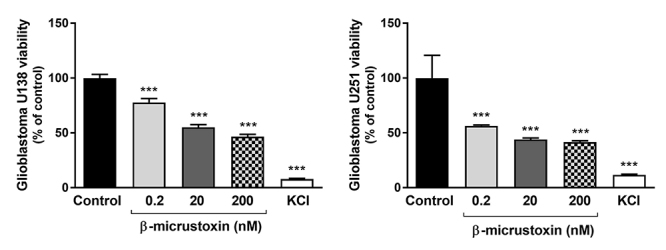
Viability of U138 and U251 glioblastoma tumor cells incubated in the absence (control) or presence of β-micrustoxin (0.2, 2, 200 nM). KCl (250 mM) represents positive control. One-way ANOVA and Bonferroni *post hoc* test: ***p < 0.001 in relation to the respective control group. N = 6-8/group, two full experimental blocks.

Glioblastoma tumor cells U138 and U251 also had their viability reduced after 24 h of incubation with β-micrustoxin at all the concentrations analyzed (Figure 4). 

### Effects of β-micrustoxin on cell proliferation

**Figure 5.**
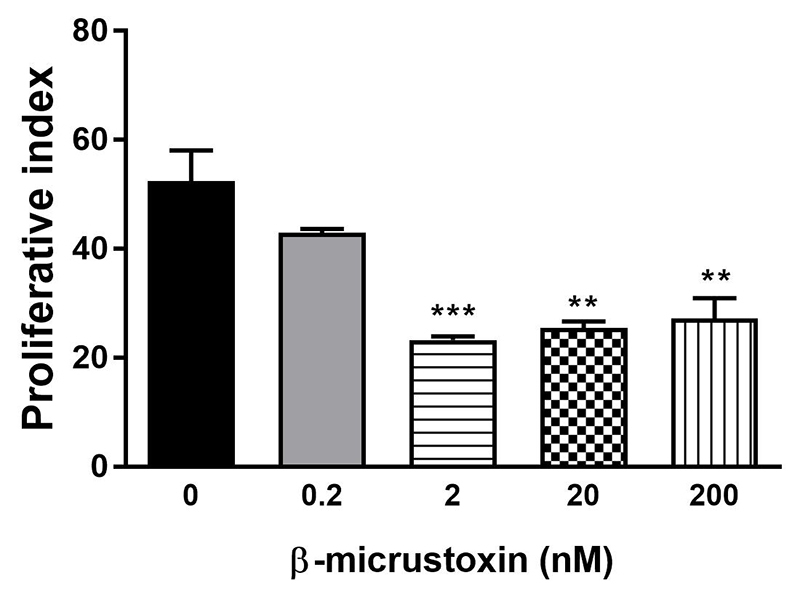
Proliferative index of cultured astrocytes incubated in the absence (control) or presence of β-micrustoxin (0.2, 2, 20, 200 nM) for 24 h evaluated by fluorescence of the carboxyfluorescein diacetate succinimidyl ester (CSFE) and flow cytometry. One-way ANOVA: p < 0.01; Bonferroni *post-hoc* test: **p < 0.01; ***p < 0.001 vs. control. N = 5/group.

Cell proliferation expressed by proliferative index was reduced after incubation of cultured astrocytes with β-micrustoxin (2, 20 and 200 nM) for 24 h (control = 52.46 ± 5.62; 0.2 nM toxin = 43.01 ± 0.71; 2 nM toxin = 23.31 ± 0.65; 20 nM toxin = 25.51 ± 1.17; 200 nM toxin = 27.23 ± 3.74) (Figure 5).

### Effects of β-micrustoxin on the distribution of cell cycle phases

**Figure 6.**
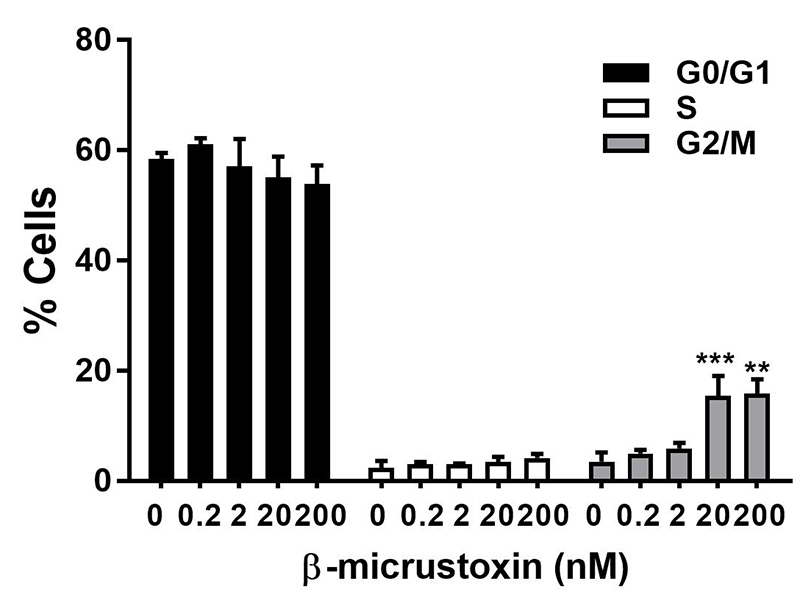
Distribution of cell cycle phases of cultured astrocytes incubated in the absence (control) or presence of β-micrustoxin (0.2, 2, 20, 200 nM) for 24 h. Phases G0/G1, S-synthesis, G2/M are expressed as a percentage. Two-way ANOVA; Bonferroni *post-hoc* test: **p < 0.01; ***p < 0.001 vs. control, N = 4/group.

The analysis of the cell cycle phases, G0 (first gap), S (DNA synthesis) and G2/M (second gap), revealed a redistribution of the phases with an increase in the G2/M phase (G2/M phase: Control = 3.58 ± 1.64; 0.2 nM toxin = 5.00 ± 0.72; 2 nM toxin = 5.99 ± 1.00; 20 nM toxin = 15.52 ± 3.58; 200 nM toxin = 15.91 ± 2.55) induced by β-micrustoxin (20 and 200 nM) without significant alterations in G0 or S phases (G0 phase: control = 58.53 ± 1.01; 0.2 nM = 61.15 ± 1.05; 2 nM = 57.19 ± 4.88; 20 nM = 55.18 ± 3.68; 200 nM = 53.91 ± 3.36; S phase: control = 2.42 ± 1.26; 0.2 nM = 3.12 ± 0.38; 2 nM = 3.19 ± .0.01; 20 nM = 3.48 ± 0.99; 200 nM = 4.23 ± 0.72) (Figure 6). 

### Evaluation of β-micrustoxin effects on DNA fragmentation

**Figure 7.**
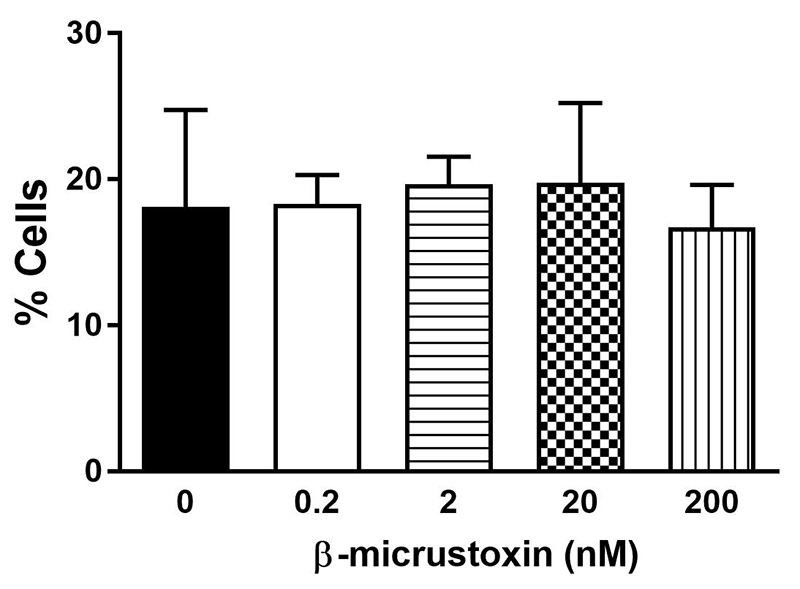
DNA fragmentation expressed by sub-G1 population of astrocytes incubated in the absence (control) or presence of β-micrustoxin (0.2, 2, 20, 200 nM) for 24 h and evaluated by flow cytometry. One-way ANOVA, p>0.05. N = 4/group.

The analysis of the sub-G1 cell population (debris and fragmented DNA) by flow cytometry suggested that there was no DNA fragmentation of astrocytes when incubated with β-micrustoxin for 24 h (control = 21.27 ± 1.55; 0.2 nM toxin = 18.33 ± 1.15; 2 nM toxin = 19.69 ± 1.33; 20 nM toxin = 19.75 ± 3.18; 200 nM toxin = 16.75 ± 1.68) (Figure 7).

### Effects of β-micrustoxin on the expression of the cell cycle regulatory proteins: p53, p21 and p27

**Figure 8.**
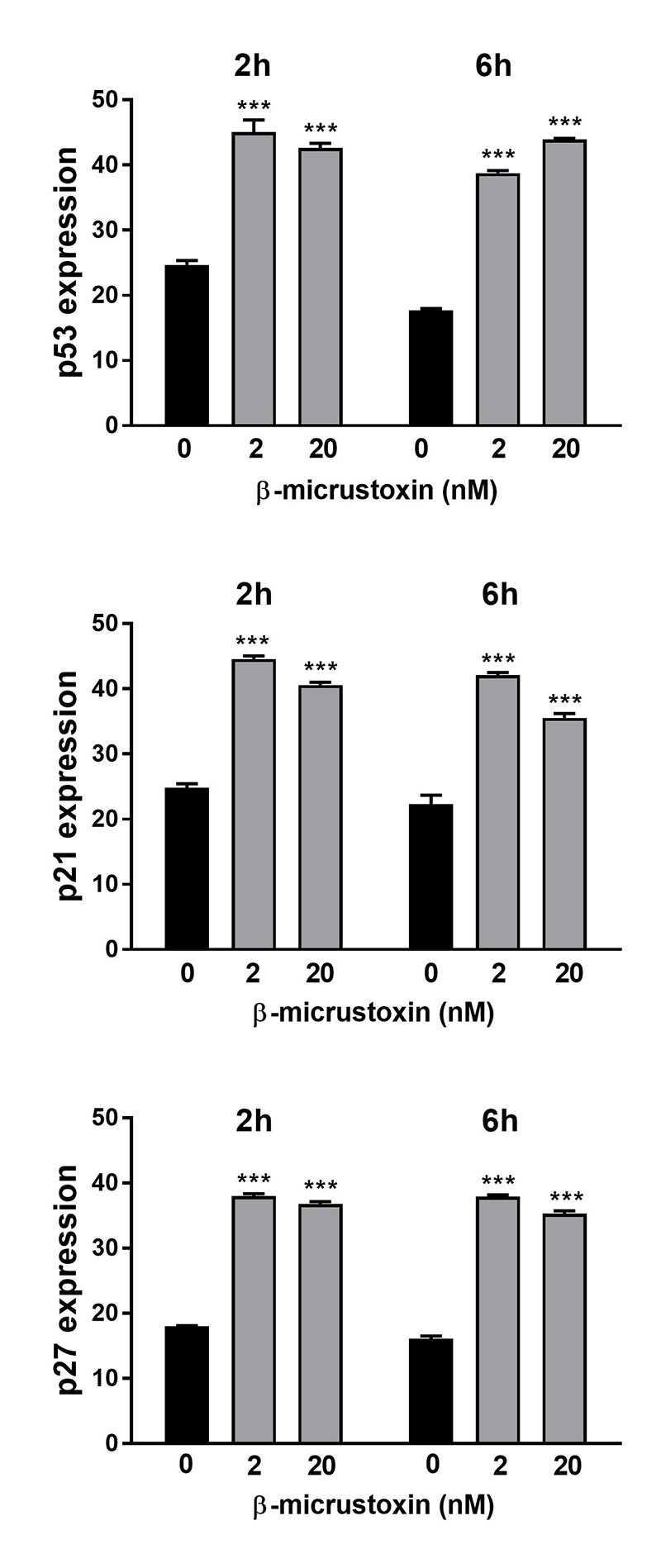
P53, p21 and p27 regulatory proteins expression in cultured astrocytes. Proteins were evaluated after incubation of astrocytes in the absence (control) or presence of β-micrustoxin (2, 20 nM) for 2 h or 6 h, by flow cytometry. Two-way ANOVA: p < 0.001; Bonferroni *post-hoc* test: ***p < 0.001 in relation to the respective control, N = 3/group.

**Figure 9.**
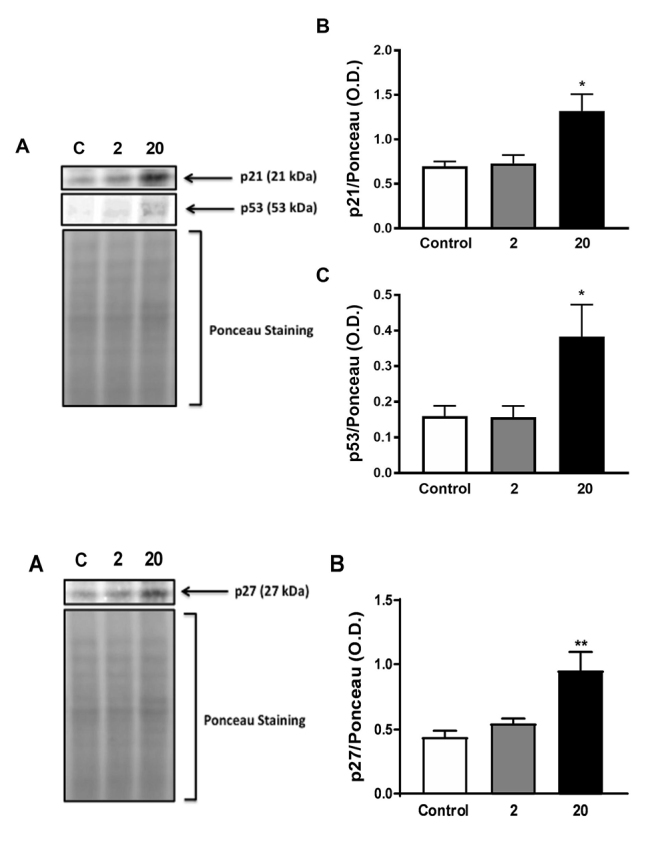
Protein expression analysis of p53, p21 and p27 in cultured astrocytes by western blotting. Astrocytes were incubated in the absence (control) or presence of β-micrustoxin (2, 20 nM) for 2 h. **(A)** Representative images of immunobloting. **(B)** and **(C)** Quantification by Ponceau analysis of p21, p27 and p53. One-way ANOVA; Bonferroni *post-hoc* test: *p < 0.05, **p < 0.01 vs. control. N = 4/group.

The expression of a transcription factor involved in cell fate, namely the tumor suppressor p53, was increased after incubation of astrocytes with β-micrustoxin (2 and 20nM) for 2 h or 6 h when analyzed by flow cytometry (Figure 8). Western blotting analysis confirmed these findings for 20 nM β-micrustoxin after 2 h of incubation (Figure 9). 

The expression of p21 and p27, inhibitory proteins of the cyclin-CDK complex, was also increased after astrocytes incubation with β-micrustoxin (2 and 20nM) for 2 h or 6 h, as shown by flow cytometry, and the same was observed after 2h of 20 nM β-micrustoxin incubation when western blotting methodology was used for analysis (Figures 8 and 9).

### p53 immunocytochemistry

**Figure 10.**
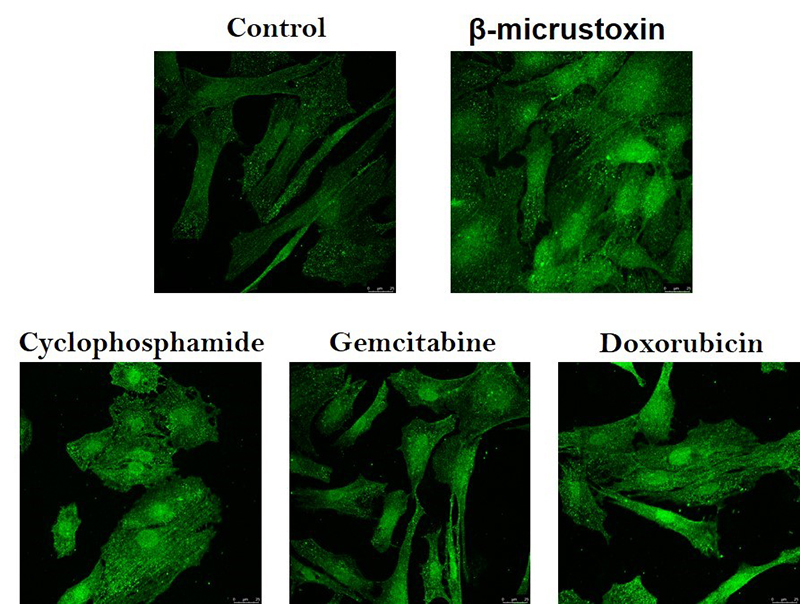
Photomicrographs of p53 immunocytochemistry in cultured astrocytes. Astrocytes were incubated in the absence (control) or presence of 2 nM β-micrustoxin, 1 mM cyclophosphamide, 0.1 µM gemcitabine or 1 µM doxorubicin (the last three are positive controls) for 2 h. The fluorescence intensification in the nuclei induced by all substances is shown. Amplification: 63x.

To illustrate the p53 activation, an immunocytochemistry analysis of p53 was performed. The photomicrographs show the intensification of fluorescence in the nucleus after 20 nM β-micrustoxin exposure for 2 h as well as after incubation with the positive controls: the chemotherapy drugs cyclophosphamide (1 mM), gemcitabine (0,1 µM) and doxorubicin (1 µM) (Figure 10). This is an indication that the p53 protein was translocated to the nucleus, which is its site of action. 

### Evaluation of TNF-α release and its involvement in the β-micrustoxin anti-proliferative effect

**Figure 11.**
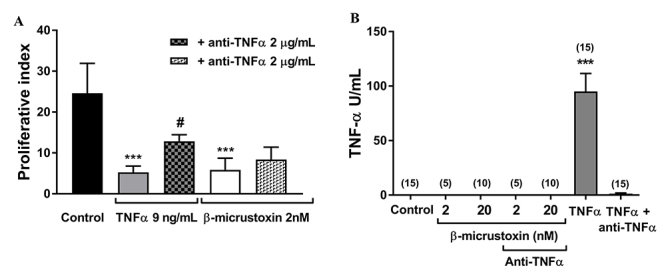
TNF-α is not involved in the reduction of astrocyte proliferation by β-micrustoxin. **(A)** Proliferative index of cultured astrocytes incubated in the absence (control) or presence of 2 nM β-micrustoxin or 9 ng/mL TNF-α and associated or not with 2 µg/mL anti-TNFα. One-way ANOVA: p < 0.0001; Bonferroni *post-hoc* test: ***p < 0.001 in relation to control group; #p < 0.05 in relation to TNFα. N = 4/group. **(B)** TNF-α released by cultured astrocytes was evaluated by the fibroblast cell line L929 assay. Cultured astrocytes were incubated in the absence (control) or presence of 2 or 20 nM β-micrustoxin or 9 ng/mL TNF-α and associated or not with 2 µg/mL anti-TNFα for 5 h. L929 cells were incubated with the astrocyte supernatant and TNF-α was quantified. One-way ANOVA; Bonferroni *post-hoc* test: ***p < 0.001. N is expressed in parentheses; two full experimental blocks.

The anti-TNFα antibody did not reverse the effect induced by the toxin on cell proliferation (Figure 11A). Thus, it is possible to infer that TNF-α is not involved in the anti-proliferative effect of β-micrustoxin in astrocytes. However, the recombinant TNF-α on its own significantly reduced the astrocyte proliferation while the antibody anti-TNF-α antagonized this effect, thus demonstrating its efficacy. 

Corroborating these data, TNF-α release by astrocytes was not observed when incubated with β-micrustoxin for 5 h or 24 h (data not shown), as evaluated by the sensitivity of the fibroblast cell line L929 (Figure 11B). The controls of this experiment using TNF-α and anti-TNF-α showed the efficiency of the analytic method using the L929 cell model because it allowed detection of the recombinant TNF-α and revealed the antagonism of the anti-TNF-α antibody. 

## Discussion

β-micrustoxin is a toxin isolated from *Micrurus lemniscatus* venom. Its PLA_2_ activity and its molecular mass (13,568 Da) have already been reported [45]. This toxin, previously denominated Mlx-9 [34, 45], was renamed β-micrustoxin in the present work for acting as a β-neurotoxin at neuronal terminals and due to its origin from *Micrurus lemniscatus* venom. 

After performing a venomic study of venoms from 6 *Micrurus* taxa, Aird *et al.* [7] found 121 new phospholipase A2 sequences, one of which was the transcript ID DN112835_C3_g9_i1/m.9019. In the present work, through proteomic analysis, the similarity between the β-micrustoxin fraction and this transcript was observed. Unfortunately, the sequence of this transcript is incomplete, and it was not added to the UniProtKB database. The PLA_2_ transcript ID DN112835_C3_g9_i1/m.9019 has an aspartate at the position 49 that is fundamental for its catalytic activity and Ca^2+^-binding. Furthermore, the presence of histidine at position 48 and tyrosine at position 52 is characteristic of the active site [55]. The PLA_2_s from the Elapidae family belong to the IA group of sPLA_2_s and present structural characteristics that differ from the IIA group of sPLA_2_s, such as the Elapidae loop.

This sPLA_2_, β-micrustoxin, comes from the venom of the coral snake that inhabits the northeastern state of Bahia. In this venom, there was a predominance of PLA_2_s corroborating Sanz *et al*. [11], who reported that *Micrurus* venoms from the state of Bahia and other northeastern states have PLA_2_ predominance. The similarity between β-micrustoxin and a PLA_2_ of *M. l. lemniscatus* suggests that the venom used in the present study could originate from this subspecies [7]. 

The pharmacological approach with the objective of characterizing the mechanism of action of β-micrustoxin was performed on cultured astrocytes and human glioblastoma tumor cells (U138 and U251 cells). β-micrustoxin reduced the viability of astrocytes as well as of the glioblastoma tumor cells. These U138 and U251 glioblastoma tumor cells originated from human astrocytes and are extremely aggressive tumors classified as grade IV.

The reduction of astrocyte viability was shown to be related to an interference with the cell proliferation process, as evidenced by a reduction in the proliferation index that express the number of cell divisions. Moreover, in these cells, the reduction of viability was not intense, in contrast to what was previously observed in hippocampal neurons [34]. As to concentration and incubation time under the same experimental conditions, neurons showed cell death while astrocytes did not. In accordance with our results, Herkert *et al*. [18] did not observe the occurrence of cell death in astrocytes incubated with β-bungarotoxin differently from the previously observed cell death in neurons. 

The predominant and relevant effect of β-micrustoxin on astrocytes was the inhibition of cell proliferation associated with a redistribution of the cell cycle phases together with an increase in the G2/M phase. This phase represents a checkpoint after the mitosis process, enabling the activation of repair mechanisms to correct possible errors in the cell division process. Activation of tumor suppressor p53 is an important pathway involved in the cell cycle progression. The magnitude of p53 expression represents a decision point for the cellular response, which can be apoptosis, cell cycle arrest, senescence, DNA repair, cell metabolism or autophagy [56-58]. P53 is a transcription factor that is considered the “guardian of the genome” since it coordinates the cellular response to various factors of cellular stress.

The p53 transcription factor seems to regulate the cell cycle by the activation of the cyclin-dependent kinase inhibitors p21 and p27 [59-61]. These proteins bind to cyclin-CDk complexes and prevent the progression of the cell cycle, thus interfering with the process of cell proliferation. Increases in the p53, p21 and p27 proteins were verified in the present work by different methodologies. Furthermore, the translocation of p53 to the nucleus was demonstrated, evidencing the activation process. P53 is constantly subjected to proteasomal proteolysis but when phosphorylated it translocates to the nucleus, activating gene transcription of, among others, p21 and p27 genes [56, 57, 61]. So far, one article reports the p53 mediation of the senescence effects of PLA_2_ [28], although the PLA_2_R also induces senescence by the activation of p53 [25].

The cytokine TNF-α, a tumor necrosis factor, not only is an important gliotransmitter released by astrocytes but also could be a mediator of both senescence and apoptosis [40, 62]. TNF-α is known to induce cell death and to control cell proliferation within certain limits, preventing or controlling the genesis of tumor cells [63]. The possibility that TNF-α could be involved in the β-micrustoxin effects was investigated. The results showed that TNF-α did not mediate the alterations induced by β-micrustoxin on astrocyte proliferation and TNF-α was not even released by astrocytes incubated with β-micrustoxin. These data are in agreement with those obtained using an inhibitor of the enzyme TACE (enzyme that promotes the release of TNF-α), showing that it did not reverse the effects of β-micrustoxin on the reduction of astrocyte viability (data not shown).

Pro- and antitumor actions of sPLA_2_ are described in the literature. An sPLA_2_ with antitumor activity was found in the snake venoms from *Bothrops*, *Crotalus* and *Naja* genus [29-33]. Some studies correlate the sPLA_2_ antitumor activity with the expression of the PLA_2_ receptor (PLA_2_R) in tumor cells and state that the ectopic expression of the PLA_2_R in PLA_2_R1-negative breast cancer cell lines strongly induces cancer cell death [26]. 

The human glioblastomas, U251 and U138 cells, with a high degree of malignancy, were sensitive to β-micrustoxin. It needs to be determined whether this effect is due to a process of cell death or to a reduction in proliferation associated or not with senescence. Likewise, it would be interesting to verify the presence of the PLA_2_R in these glioblastoma tumor cells and whether it is associated with the sensitivity of these cells to β-micrustoxin.

## Conclusion

In summary, β-micrustoxin, an sPLA_2_ from *Micrurus lemniscatus* venom, is an inhibitor of astrocyte proliferation, an effect that seems to be mediated by cell cycle regulatory proteins, the tumor suppressor p53 and two cyclin-dependent kinase inhibitors, p21 and p27. Cell cycle arrest was evidenced by a redistribution of the cell cycle phases with a G2/M increase. Glioblastoma tumor cells had their viability also reduced by β-micrustoxin. This sPLA_2_ has similarities with an sPLA_2_ from *Micrurus lemniscatus lemniscatus*, described by Aird *et al*. [7] (transcript ID: DN112835_C3_g9_i1/m.9019). The identification of an sPLA_2_ that inhibits the astrocyte proliferation process and reduces tumor-cell viability would open up novel perspectives in searching for new molecules that could potentially promote tumor growth arrest.

## Supplementary material

The following online material is available for this article:

Additional file 1. Chymotryptic peptides identified for the transcript ID sequence DN112835_C3_g9_i1/m.9019n. **(A)** Annotated spectrum with alignment of the ion 446.71 2+ and its **(B)** ion table and error map. **(C)** Annotated spectrum with alignment of the ion 637.31 2+ and its **(D)** ion table and error map. **(E)** Annotated spectrum with alignment of the ion 826.44 1+ and its **(F)** ion table and error map. **(G)** Annotated spectrum with alignment of the ion 945.42 2+ and its **(H)** ion table and error map. **(I)** Annotated spectrum with alignment of the ion 1016.42 1+ and its **(J)** ion table and error map.

Additional file 2. Tryptic peptides identified for the transcript ID sequence DN112835_C3_g9_i1/m.9019. **(A)** Annotated spectrum with alignment of the ion 812.46 1+ and its **(B)** ion table and error map. **(C)** Annotated spectrum with alignment of the ion 569.24 2+ and its **(D)** ion table and error map.

Additional file 3. Tryptic peptide identified for LAA24104.1 protein. **(A)** Annotated spectrum with alignment of the ion 677.81 2+ and its **(B)** ion table and error map.
