# Supplementary material for: β-micrustoxin (Mlx-9), a PLA2 from Micrurus lemniscatus snake venom: biochemical characterization and anti-proliferative effect mediated by p53
Source: J Venom Anim Toxins Incl Trop Dis. 2022 Apr 11;28:e20210094. doi: 10.1590/1678-9199-JVATITD-2021-0094 (PMC9008913; doi:10.1590/1678-9199-JVATITD-2021-0094)
Supplement: Additional file 1. [file 1678-9199-jvatitd-28-e20210094-s1.pdf]

J Venom Anim Toxins incl Trop Dis
https://doi.org/10.1590/1678-9199-JVATITD-2021-0094

Supplementary Material to "β-micrustoxin (Mlx-9), a PLA_2 from *Micrurus lemniscatus* snake venom: biochemical characterization and anti-proliferative effect mediated by p53"

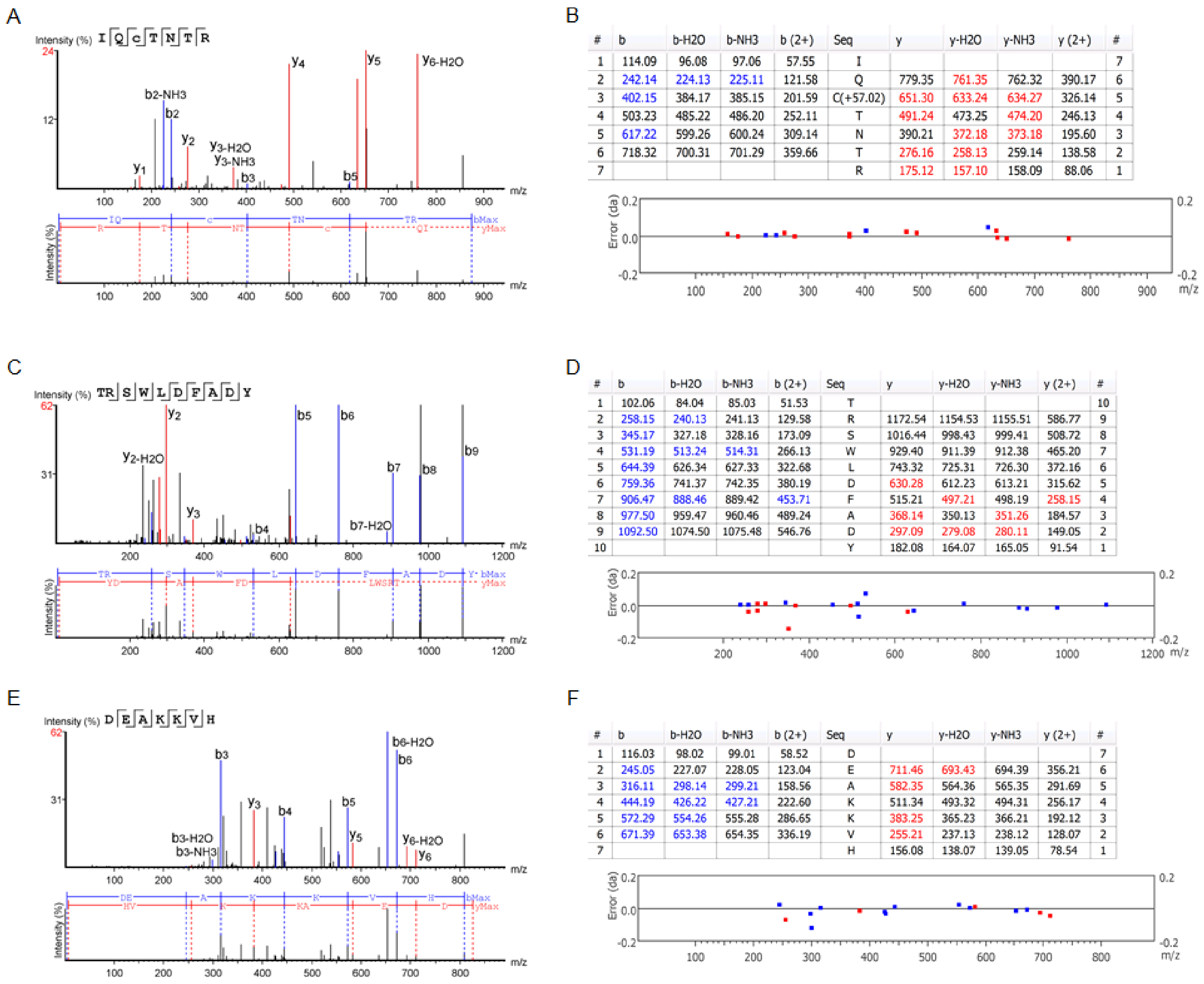

B

#	b	b-H2O	b-NH3	b (2+)	Seq	y	y-H2O	y-NH3	y (2+)	#
1	114.09	96.08	97.06	57.55	I					7
2	242.14	224.13	225.11	121.58	Q	779.35	761.35	762.32	390.17	6
3	402.15	384.17	385.15	201.59	C(+57.02)	651.30	633.24	634.27	326.14	5
4	503.23	485.22	486.20	252.11	T	491.24	473.25	474.20	246.13	4
5	617.22	599.26	600.24	309.14	N	390.21	372.18	373.18	195.60	3
6	718.32	700.31	701.29	359.66	T	276.16	258.13	259.14	138.58	2
7					R	175.12	157.10	158.09	88.06	1

D

#	b	b-H2O	b-NH3	b (2+)	Seq	y	y-H2O	y-NH3	y (2+)	#
1	102.06	84.04	85.03	51.53	T					10
2	258.15	240.13	241.13	129.58	R	1172.54	1154.53	1155.51	586.77	9
3	345.17	327.18	328.16	173.09	S	1016.44	998.43	999.41	508.72	8
4	531.19	513.24	514.31	266.13	W	929.40	911.39	912.38	465.20	7
5	644.39	626.34	627.33	322.68	L	743.32	725.31	726.30	372.16	6
6	759.36	741.37	742.35	380.19	D	630.28	612.23	613.21	315.62	5
7	906.47	888.46	889.42	453.71	F	515.21	497.21	498.19	258.15	4
8	977.50	959.47	960.46	489.24	A	368.14	350.13	351.26	184.57	3
9	1092.50	1074.50	1075.48	546.76	D	297.09	279.08	280.11	149.05	2
10					Y	182.08	164.07	165.05	91.54	1

F

#	b	b-H2O	b-NH3	b (2+)	Seq	y	y-H2O	y-NH3	y (2+)	#
1	116.03	98.02	99.01	58.52	D					7
2	245.05	227.07	228.05	123.04	E	711.46	693.43	694.39	356.21	6
3	316.11	298.14	299.21	158.56	A	582.35	564.36	565.35	291.69	5
4	444.19	426.22	427.21	222.60	K	511.34	493.32	494.31	256.17	4
5	572.29	554.26	555.28	286.65	K	383.25	365.23	366.21	192.12	3
6	671.39	653.38	654.35	336.19	V	255.21	237.13	238.12	128.07	2
7					H	156.08	138.07	139.05	78.54	1

G

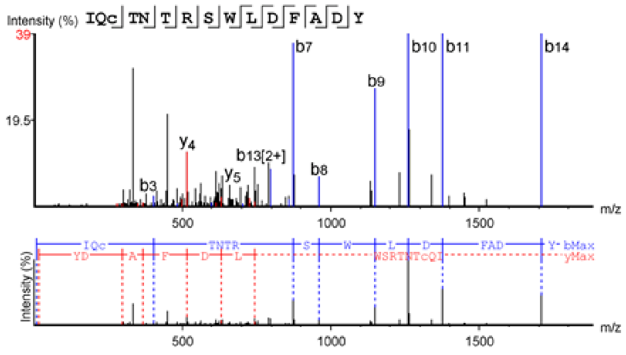

H

#	b	b-H2O	b-NH3	b (2+)	Seq	y	y-H2O	y-NH3	y (2+)	#
1	114.09	96.08	97.06	57.55	I					15
2	242.15	224.14	225.12	121.58	Q	1776.76	1758.75	1759.74	888.88	14
3	402.20	384.17	385.15	201.59	C(+57.02)	1648.71	1630.70	1631.68	824.85	13
4	503.23	485.22	486.20	252.11	T	1488.68	1470.66	1471.65	744.84	12
5	617.27	599.23	600.24	309.14	N	1387.63	1369.62	1370.60	694.31	11
6	718.32	700.31	701.34	359.66	T	1273.58	1255.57	1256.56	637.29	10
7	874.42	856.41	857.41	437.71	R	1172.54	1154.53	1155.51	586.77	9
8	961.48	943.44	944.43	481.18	S	1016.44	998.43	999.41	508.72	8
9	1147.49	1129.52	1130.50	574.27	W	929.40	911.39	912.38	465.20	7
10	1260.57	1242.61	1243.59	630.81	L	743.36	725.37	726.30	372.16	6
11	1375.57	1357.63	1358.62	688.32	D	630.26	612.24	613.21	315.62	5
12	1522.71	1504.70	1505.68	761.86	F	515.18	497.18	498.19	258.11	4
13	1593.75	1575.74	1576.72	797.39	A	368.20	350.14	351.12	184.57	3
14	1708.80	1690.76	1691.75	854.89	D	297.16	279.17	280.08	149.05	2
15					Y	182.08	164.07	165.05	91.54	1

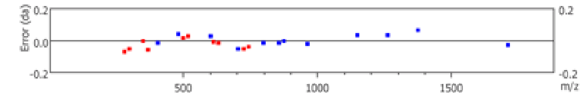

I

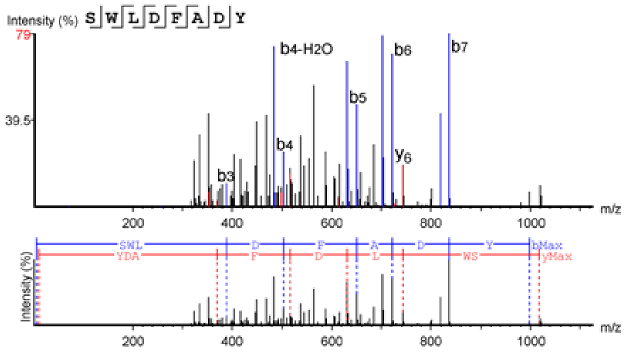

J

#	b	b-H2O	b-NH3	b (2+)	Seq	y	y-H2O	y-NH3	y (2+)	#
1	88.04	70.03	71.03	44.52	S					8
2	274.12	256.11	256.90	137.56	W	929.40	911.39	912.38	465.20	7
3	387.20	369.19	370.18	194.10	L	743.35	725.30	726.30	372.16	6
4	502.21	484.21	485.20	251.62	D	630.23	612.25	613.21	315.62	5
5	649.32	631.29	632.30	325.15	F	515.20	497.20	498.23	258.11	4
6	720.36	702.35	703.36	360.67	A	368.17	350.13	351.18	184.57	3
7	835.37	817.36	818.37	418.18	D	297.11	279.10	280.08	149.05	2
8					Y	182.08	164.07	165.05	91.54	1

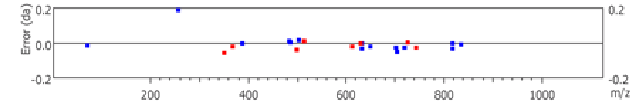

Additional file 1. Chymotryptic peptides identified for the transcript ID sequence DN112835_C3_g9_i1/m.9019n. **(A)** Annotated spectrum with alignment of the ion 446.71 2+ and its **(B)** ion table and error map. **(C)** Annotated spectrum with alignment of the ion 637.31 2+ and its **(D)** ion table and error map. **(E)** Annotated spectrum with alignment of the ion 826.44 1+ and its **(F)** ion table and error map. **(G)** Annotated spectrum with alignment of the ion 945.42 2+ and its **(H)** ion table and error map. **(I)** Annotated spectrum with alignment of the ion 1016.42 1+ and its **(J)** ion table and error map.
