# Supplementary material for: β-micrustoxin (Mlx-9), a PLA2 from Micrurus lemniscatus snake venom: biochemical characterization and anti-proliferative effect mediated by p53
Source: J Venom Anim Toxins Incl Trop Dis. 2022 Apr 11;28:e20210094. doi: 10.1590/1678-9199-JVATITD-2021-0094 (PMC9008913; doi:10.1590/1678-9199-JVATITD-2021-0094)
Supplement: Additional file 2. [file 1678-9199-jvatitd-28-e20210094-s2.pdf]

J Venom Anim Toxins incl Trop Dis
https://doi.org/10.1590/1678-9199-JVATITD-2021-0094

Supplementary Material to "β-micrustoxin (Mlx-9), a PLA_2 from *Micrurus lemniscatus* snake venom: biochemical characterization and anti-proliferative effect mediated by p53"

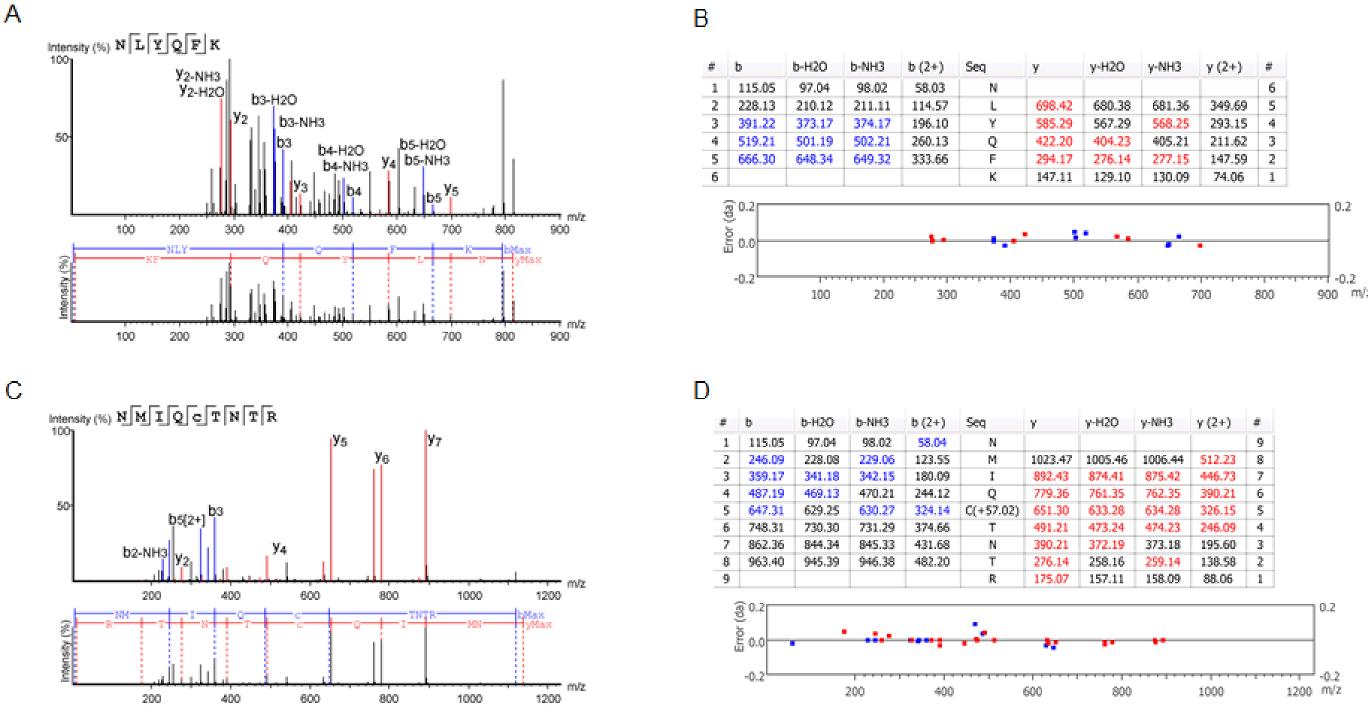

B

#	b	b-H2O	b-NH3	b (2+)	Seq	y	y-H2O	y-NH3	y (2+)	#
1	115.05	97.04	98.02	58.03	N					6
2	228.13	210.12	211.11	114.57	L	698.42	680.38	681.36	349.69	5
3	391.22	373.17	374.17	196.10	Y	585.29	567.29	568.25	293.15	4
4	519.21	501.19	502.21	260.13	Q	422.20	404.23	405.21	211.62	3
5	666.30	648.34	649.32	333.66	F	294.17	276.14	277.15	147.59	2
6					K	147.11	129.10	130.09	74.06	1

D

#	b	b-H2O	b-NH3	b (2+)	Seq	y	y-H2O	y-NH3	y (2+)	#
1	115.05	97.04	98.02	58.04	N					9
2	246.09	228.08	229.06	123.55	M	1023.47	1005.46	1006.44	512.23	8
3	359.17	341.18	342.15	180.09	I	892.43	874.41	875.42	446.73	7
4	487.19	469.13	470.21	244.12	Q	779.36	761.35	762.35	390.21	6
5	647.31	629.25	630.27	324.14	C(+57.02)	651.30	633.28	634.28	326.15	5
6	748.31	730.30	731.29	374.66	T	491.21	473.24	474.23	246.09	4
7	862.36	844.34	845.33	431.68	N	390.21	372.19	373.18	195.60	3
8	963.40	945.39	946.38	482.20	T	276.14	258.16	259.14	138.58	2
9					R	175.07	157.11	158.09	88.06	1

Additional file 2. Tryptic peptides identified for the transcript ID sequence DN112835_C3_g9_i1/m.9019. **(A)** Annotated spectrum with alignment of the ion 812.46 1+ and its **(B)** ion table and error map. **(C)** Annotated spectrum with alignment of the ion 569.24 2+ and its **(D)** ion table and error map.
