# Supplementary material for: β-micrustoxin (Mlx-9), a PLA2 from Micrurus lemniscatus snake venom: biochemical characterization and anti-proliferative effect mediated by p53
Source: J Venom Anim Toxins Incl Trop Dis. 2022 Apr 11;28:e20210094. doi: 10.1590/1678-9199-JVATITD-2021-0094 (PMC9008913; doi:10.1590/1678-9199-JVATITD-2021-0094)
Supplement: Additional file 3. [file 1678-9199-jvatitd-28-e20210094-s3.pdf]

J Venom Anim Toxins incl Trop Dis
https://doi.org/10.1590/1678-9199-JVATITD-2021-0094

Supplementary Material to "β-micrustoxin (Mlx-9), a PLA_2 from *Micrurus lemniscatus* snake venom: biochemical characterization and anti-proliferative effect mediated by p53"

A

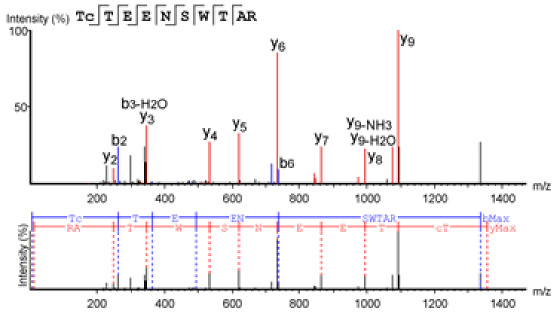

B

#	b	b-H2O	b-NH3	b (2+)	Seq	y	y-H2O	y-NH3	y (2+)	#
1	102.06	84.04	85.03	51.53	T					11
2	262.08	244.08	245.06	131.54	C(+57.02)	1253.52	1235.51	1236.49	627.26	10
3	363.08	345.13	346.11	182.07	T	1093.46	1075.42	1076.44	547.25	9
4	492.29	474.20	475.15	246.59	E	992.44	974.40	975.42	496.72	8
5	621.22	603.21	604.19	311.11	E	863.41	845.36	846.36	432.20	7
6	735.37	717.33	718.35	368.13	N	734.39	716.40	717.33	367.68	6
7	822.29	804.28	805.27	411.65	S	620.32	602.30	603.29	310.66	5
8	1008.37	990.36	991.35	504.69	W	533.25	515.23	516.23	267.14	4
9	1109.42	1091.41	1092.39	555.21	T	347.20	329.20	330.18	173.96	3
10	1180.46	1162.45	1163.43	590.73	A	246.16	228.15	229.06	123.58	2
11					R	175.12	157.11	158.09	88.06	1

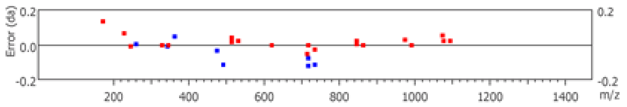

Additional file 3. Tryptic peptide identified for LAA24104.1 protein. **(A)** Annotated spectrum with alignment of the ion 677.81 2+ and its **(B)** ion table and error map.
